# High‐fat diet‐induced hypertension and autonomic imbalance are associated with an upregulation of CART in the dorsomedial hypothalamus of mice

**DOI:** 10.14814/phy2.12811

**Published:** 2016-06-06

**Authors:** Laiali J. Chaar, Aline Coelho, Natalia M. Silva, William L. Festuccia, Vagner R. Antunes

**Affiliations:** ^1^ Department of Physiology and BiophysicsInstitute of Biomedical SciencesUniversity of Sao PauloSao PauloBrazil

**Keywords:** Cocaine‐ and amphetamine‐regulated transcript, dorsomedial hypothalamus, hypertension, obesity, sucrose, sympathetic nervous system

## Abstract

We evaluated herein whether diet‐induced obesity alters sympathovagal balance, blood pressure, and neuropeptides levels at the hypothalamus and brainstem of mice. Male C57BL6J mice fed with a high‐fat (HFD) or a high‐fat high‐sucrose (HFHSu), or a regular chow diet (C) for 8 weeks were evaluated for metabolic parameters and blood pressure, the latter being performed in conscious freely moving mice. Spectral analysis from the records of systolic blood pressure (SBP) and cardiac pulse intervals (PI) was performed to analyse the autonomic balance in the cardiovascular system. HFD‐fed mice developed two distinct hemodynamic phenotypes: hypertensive mice (HFD‐H) with high systolic and diastolic BP levels and hypertension‐resistant mice (HFD‐R) whose BP levels were similar to C group. Spectral analysis of SBP and PI variabilities indicate that the low‐frequency (LF)/high‐frequency (HF) ratio, which is an index of sympathovagal balance, is higher in HFD‐H compared to HFD‐R. Along with hypertension and higher LF/HF ratio, HFD‐H mice presented increased hypothalamic mRNA levels of cocaine‐ and amphetamine‐regulated transcript (CART), and increased CART‐positive neurones in the dorsomedial hypothalamus (DMH) by high‐fat diet when compared to C group. Despite developing obesity to similar levels than HFD feeding, intake of a HFHSu was not associated with hypertension in mice neither CART levels increase. Collectively, our main findings indicate that high‐fat diet induced‐hypertension and autonomic imbalance are associated to an upregulation of CART levels in the DMH of mice.

## Introduction

Obesity is a pandemic disorder and a risk factor for many diseases including diabetes, cancer, and cardiovascular maladies such as hypertension (Meigs et al. 1997; Montague and O'Rahilly 2000; WHO, 2010). Despite much work in this field, the mechanisms through which excessive fat accumulation results in an increased incidence of hypertension are still a matter of debate.

Hyperactivity of the sympathetic nervous system (SNS), a remarkable feature of obesity, has been implicated in the genesis and maintenance of obesity‐associated hypertension (Esler 1995; Reaven 2002; Hall 2003; Grassi et al. 2004; Bergman et al. 2007; Hall et al. 2010). SNS activity is constantly modulated by a complex interaction of neurotransmitters at the hypothalamic and brainstem autonomic nuclei level that controls not only blood pressure levels, but also energy balance and body weight.

Of particular interest, the dorsomedial hypothalamus (DMH) is an important center involved in autonomic and neuroendocrine responses, such as cardiac sympathetic hyperactivity, hypertension, feeding behaviour, hyperventilation, and increased locomotor activity (Yardley and Hilton 1986; Fontes et al. 2001; Bellinger and Bernardis 2002; DiMicco et al. 2002; Zaretskaia et al. 2002; Cao et al. 2004). In addition to the DMH, the arcuate nucleus of hypothalamus (Arc) also contains two subtypes of neurones: (1) orexigenic, which stimulates food intake and inhibit energy expenditure by secreting agouti‐related protein (AgRP) and/or neuropeptide Y (NPY); and (2) anorexigenic neurones that inhibit food intake and stimulate energy expenditure by secreting Pro‐opiomelanocortin (POMC) and/or cocaine‐ and amphetamine‐regulated transcript (CART) (Schwartz et al. 2000).

Cocaine‐ and amphetamine‐regulated transcript is coexpressed with NPY in the DMH and its mRNA levels are upregulated after 10 weeks of high‐fat diet feeding. Noteworthy, some of these DMH CART‐positive neurones are orexigenic (Lee et al. 2013a). Moreover, CART plays an important role on the autonomic nervous system control, since intracerebroventricular injection of CART in conscious rabbits evokes a significant increase in blood pressure and heart rate (Matsumura et al. 2001).

Thus, here we hypothesize that intake of a high‐fat diet leads to changes in neuropeptides levels within autonomic nuclei in the hypothalamus and brainstem that could contribute to the development of obesity‐associated sympathetic hyperactivity and hypertension. To test this, we screened the hypothalamus and brainstem of mice‐induced obese by two different regimens, namely intake of a high‐fat diet (HFD) or a high‐fat diet combined with a 20% sucrose in water (HFHSu), for neuropeptides that might be involved in the obesity‐associated hypertension and autonomic imbalance. We have found that intake of HFD, but not HFHSu, induces autonomic imbalance and hypertension in mice, phenotypes that were associated with an upregulation of CART levels at the DMH.

## Methods

### Animals

All experimental procedures were performed in accordance with the Ethical Principles in Animal Research of the Brazilian College of Animal Experimentation and were approved by the Ethical Committee for Animal Research of ICB/USP (Protocol #026/126‐02). Male mice C57BL/6J (10 weeks of age) obtained from the Institute of Biomedical Sciences, University of Sao Paulo, were kept at a constant temperature of 23 ± 1°C, relative humidity of 50–60% and light/dark cycle (12/12 h). Mice were fed with different diets as described below and drinking water ad libitum.

### Diet

Mice were fed with either a regular chow diet (C; Nuvilab^®^ CR1, Sogorb Inc, 66% carbohydrates, 22% protein, 7% fibers, and 5% lipids, in %Kcal, *n* = 8), or a high‐fat diet [HFD; 13% carbohydrates, 20% protein, 7% fibers, and 60% lipids, in %Kcal; adapted from a previous study by Reeves (1997), *n* = 24] or high‐fat high‐sucrose in association with 20% sucrose in the drinking water (HFHSu, *n* = 11) during 8 weeks. Body weight, food, and water intake were assessed weekly.

### Glucose tolerance test

At 8th week, a glucose tolerance test (GTT) was performed in C (*n* = 8), HFD (*n* = 24), and HFHSu (*n* = 11) mice. After 6 h of fasting, mice received an intraperitoneal injection of glucose (1 g/kg) and blood glucose levels were determined from an incision in the tail using Accu‐Check Compact glucometer (Roche Diagnostics, Basel, Switzerland) at 0, 15, 30, 45, 60, 90, and 120 min after injection.

### Adiposity index

Animals were killed under anaesthesia with isoflurane (5% in O_2_‐air inspired) between 8 and 10 am after overnight fasting followed by 2 h with free access to food to standardization of food intake. The sum of the epididymal, retroperitoneal, and inguinal adipose tissues mass was considered as an adiposity index in C (*n* = 8), HFD (*n* = 24), and HFHSu (*n* = 11).

### Plasmatic parameters

Plasma levels of leptin, insulin, interleukin‐6 (IL‐6), and resistin were evaluated by ELISA (Millipore, Billerica, MA) in refed conditions in C (*n* = 8), HFD (*n* = 24) and HFHSu (*n* = 11) mice. Plasmatic‐free fatty acid (Wako Diagnostics, Mountain View, CA) and triglycerides (LabTest, Lagoa Santa, Minas Gerais, Brazil) concentrations were measured following the manufacturer's recommendations.

### Hemodynamic recordings

After 8 weeks, femoral artery catheterization was performed under anesthesia with isoflurane (5% in O_2_‐air inspired) in C (*n* = 8), HFD (*n* = 24), and HFHSu (*n* = 11) mice. A microrenathane catheter (0.025 mm outer diameter and 0.012 mm internal diameter) was inserted in the femoral artery, tunneled subcutaneously and exteriorized at the scapular region. Animals were housed in individual cages and allowed to recover for 1 day. Arterial pressure and heart rate (HR) were monitored in conscious freely moving mice between 8 and 10 am, by connecting the arterial catheter, previously heparinized, to a pressure transducer (Model CDX III, Cobe Laboratories, Lakewood, CO) connected to an amplifier (ML224 Quad Bridge Amp, ADInstruments, NSW, Australia) and a digital data acquisition system (PowerLab, ADInstruments). The sample rate of the hemodynamic parameters acquisition was 4 kHz. After mice acclimation to the recording room, the cardiovascular parameters were recorded for 90 min. Mean arterial pressure (MAP), systolic (SBP), and diastolic blood pressure (DBP) and spectral analysis parameters were analyzed off‐line. Beat‐by‐beat pulse interval (PI) values were generated off‐line from the pulsatile arterial pressure signal by measuring the time interval between two systolic peaks.

### Spectral analysis

Data analysis of systolic blood pressure and pulse interval (PI) variabilities were performed using the software CardioSeries (http://www.danielpenteado.com/) following the guidelines for mice as previously described (Thireau et al. 2008). A stable 10 min SBP and PI records without artifacts or large sudden blood pressure changes of each animal were used in the analysis. Beat‐by‐beat time series of SBP and PI generated from the acquisition software (Lab Chart Pro, AD Instruments) were loaded into CardioSeries software that performs time and frequency domain analysis of arterial pressure and heart rate variability. While the time domain assesses the magnitude of the temporal variations in the cardiac rhythm modulated by autonomic nervous system, that is, SBP and PI lability, the frequency domain analysis provides the spectral composition of these variations (Wang and Huang 2012). SBP and PI power spectral density were estimated by Fast Fourier transform algorithm for discrete time series. Using 20 Hz of interpolation rate, beat‐by‐beat series were divided in half‐overlapping sequential sets with 512 points. All segments were tested for stationary conditions, that is, if they had mean and covariance stable over time, by means of stationary tests (Berntson et al. 1997; van de Borne et al. 1997; Porta et al. 2004). Nonstationary data and segments with transients were not included in the power spectral density calculation. Variability index of SBP and PI lability were calculated in the time domain. The SBP variability was quantified by the standard deviation of successive SBP values (SDNN). The variability in PI was quantified by standard deviation of normal‐to‐normal beats intervals (SDNN) and by the root mean square of successive differences (RMSSD) between adjacent normal PI. Thus, for the frequency domain analysis, the SBP and PI spectra were integrated in frequency bands considering the ranges of mice HR and breathing frequencies: very low‐frequency (VLF, 0.00–0.15 Hz), low‐frequency (LF, 0.15–1.50 Hz), and high‐frequency (HF, 1.50–5 Hz) bands as previously described (Thireau et al. 2008). Results are expressed in absolute (ms^2^ or mmHg^2^) and normalized units (nu) that is low‐frequency or high‐frequency total power in % excluding very low‐frequency band (van de Borne et al. 1997; Billman 2011). To assess the sympathovagal balance to the heart, low‐frequency/high‐frequency ratio (LF/HF) of PI variability was calculated (Montano et al. 1994).

### Quantitative PCR

Hypothalamic and brainstem messenger RNA (mRNA) levels of neuropeptides were quantified by qPCR (Quantitative PCR) in the same group of mice that hemodynamic recordings, and spectral analysis were performed [C (*n* = 6), HFD (*n* = 15), and HFHSu (*n* = 10)]. All animals were killed between 8 and 10 am after overnight fasting followed by 2 h with free access to food to avoid feeding related and circadian changes in gene expression. After brain removal from the skull, a hypothalamic tissue block was dissected at the level of +0.26 to −2.30 mm from bregma and is a square between the optic chiasma and the superior cerebellar peduncle. Brainstem samples were dissected at the level of −5.80 to −8.00 mm from bregma between the inferior cerebellar peduncle and the obex. Hypothalamic and brainstem blocks were immediately frozen in liquid nitrogen and stored at −80°C. Total RNA was extracted using TRizol (Life Technologies, Carlsbad, CA) and Illustra RNAspin Mini kit (GE Healthcare Life Sciences, Amersham Place, Little Chalfont, Buckinghamshire, UK) following the manufacturer's instructions. Total RNA levels were quantified using a spectrophotometer (“Nanodrop 3300” – Thermo Scientific, Waltham, MA). cDNA synthesis was performed with Superscript Reverse Transcriptase III (Invitrogen, Carlsbad, CA) with 1 ug of total RNA. The amplification reaction was performed on rotor Gene Q (Qiagen, Venlo, the Netherlands) using SYBR Green (Sigma‐Aldrich, St. Louis, MO) and specific oligonucleotides for cocaine‐ and amphetamine‐regulated transcript (CART, forward 5′‐GTCCCACGAGAAGGAGCTGCCAA‐3′; reverse 5′‐GCCCATCCGCTCTCTGAGGGG‐3′), pro‐opiomelanocortin (POMC, forward 5′‐GCCTTTCCGCGACAGGGGTC‐3′; reverse 5′‐AAACACGGGCGTTCCAGCG‐3′), neuropeptide Y (NPY, forward 5′‐AGCCTTGTTCTGGGGGCGTT‐3′; reverse 5′‐CCCGCCACGATGCTAGGTAA‐3′) or agouti‐related protein (AgRP, forward 5′‐ACCTTAGGGAGGCACCTCATGCC–3′; reverse 5′‐GCGGAGAACGAGACTCGCGG‐3′). Ribossomal protein 36B4 (forward 5′‐ CCACTTACTGAAAAGGTCAAGGC‐3′; reverse 5′‐TGGTTGCTTTGGCGGGATTTA‐3′), was used as a reporter gene because it not changed by hypertension or obesity. The specificity of the PCR amplification was confirmed by melting curve analysis. For quantification of the results of real‐time PCR, the device software (Rotor Gene Q, Qiagen) determined threshold (Ct) (Ramakers et al. 2003). From these values, EfΔΔCt was calculated (Schefe et al. 2006). Data are expressed as the ratio between the expression of target genes and the housekeeping gene 36B4.

### Immunohistochemistry

as for the immunohistochemistry studies mice [C (*n* = 4), HFD (*n* = 4), and HFHSu (*n* = 5)] were deeply anesthetized with isoflurane (5% in O_2_‐air inspired) and intracardially perfused with saline (0.9%), followed by paraformaldehyde (PFA 4% in 0.1 mol L^−1^ phosphate buffer, pH 7.4) through a needle connected to a peristaltic pump (Cole Parmer, Chicago, IL). The brains were immediately removed, post fixed for 4 h at 4°C in 4% PFA and cryoprotected in phosphate buffer containing 20% sucrose at 4°C overnight. On the following day, brains were sectioned in the coronal plane on a freezing microtome; 30‐*μ*m‐thick sections were collected in four compartments. Tissues were stored in antifreeze solution. The sections were incubated for 40 h at 4°C with rabbit anti‐CART (55‐102) antibody (Phoenix Pharmaceutics, Mannheim, Germany) at a concentration of 1:20 000 in phosphate buffer containing 2% goat normal serum and 0.3% Triton. This antibody concentration was chosen after a previous titration with different concentrations: 1:500, 1:10 000, and 1:20 000. This antibody has been extensively used in previous work and reacts in brain nuclei confirming the presence of CART (Elias et al. 2001). After several washes in 0.02 mol L^−1^ potassium phosphate buffer (KPBS), sections were incubated for two hours in biotinylated goat antirabbit antibody (1:200, Vector Laboratories, Burlingame, CA). Sections were washed again in 0.02 mol L^−1^ and then incubated for 2 h in an avidin‐biotin complex (Vectastain Elite ABC kit, Vector Laboratories, 1:200) followed by a peroxidase reaction product, which was visualized using the glucose oxidase method with DAB (diaminobenzidine) as chromogen (Itoh et al. 1979) and mounted on gelatinized slides. Immunostaining was enhanced by soaking the slides in a solution of osmium tetroxide for 15 sec. Finally, the slides were dehydrated, cleared in xylene and covered with DPX (Sigma‐Aldrich). Sections of mice brain stained by immunoperoxidase reaction were photographed using a Zeiss Axioimager A1 (Carl Zeiss AG Corporate, Oberkochen, Germany) equipped with a digital camera of the type Zeiss HRc AxioCam (Carl Zeiss AG Corporate). Images from the hypothalamus and brainstem were taken using landmarks for the nuclei according to (Franklin and Paxinos 2008). Particularly, images throughout of the DMH were taken between bregma level −1.34 to −2.18 mm (Zhang et al. 2011). Quantitative differences in CART‐positive neurones were analyzed among groups by counting neuronal cell bodies only, not fibers (qualitative analysis), immunostained for CART.

### Statistical analysis

All results are expressed as means ± standard error of mean. Statistical analysis was performed with GraphPad Prism 5.0 software (GraphPad, La Jolla, CA). Differences were considered significant when *P *<* *0.05. One‐way analysis of variance (ANOVA) with Newman–Keuls post hoc test were used to analyse the data.

## Results

### High‐fat diet elicits metabolic and biochemical changes associated with obesity

**Figure 1:**
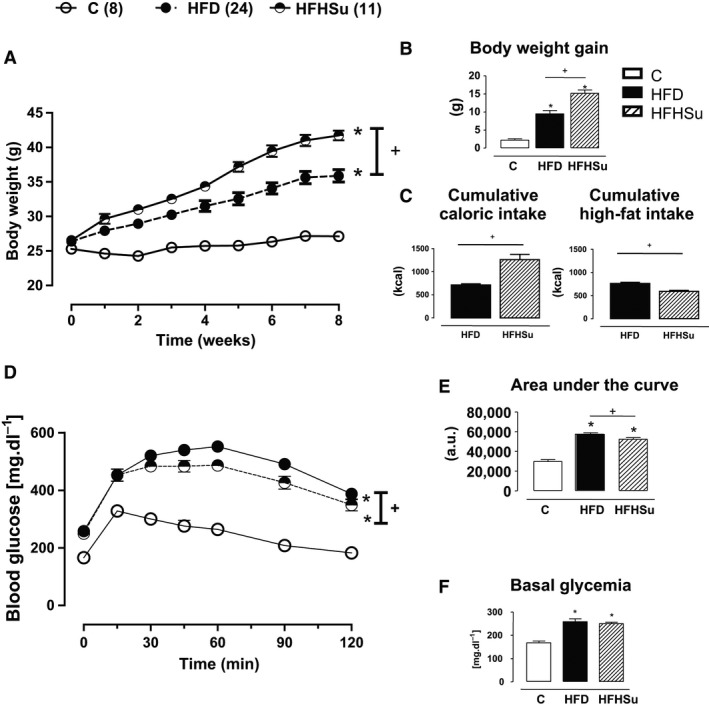
Body composition, metabolic, and biochemical parameters. (A) body weight mass (g), (B) body weight gain (g), (C) cumulative carolic intake (kcal), (D) cumulative high fat intake (kcal), (E) glucose tolerance test, (F) basal glycemia at fasting, (G) area under the curve (AUC; a.u. = arbitrary units) of the glucose tolerance test, and (H) mass (g) of epididymal adipose tissue, retroperitoneal adipose tissue, inguinal adipose tissue, adiposity and liver of mice over the 8 weeks of exposure to control (C, *n* = 8), high‐fat diet (HFD, *n* = 24) or high‐fat high‐sucrose (HFHSu, *n* = 11). *P < 0.01 versus C and ^+^ *P* < 0.01 versus HFD. One‐way ANOVA with Newman–Keuls post hoc test.

High‐fat or HFHSu diet intake induced obesity in mice as evidenced by the increased body weight (Fig. 1A and B), adiposity (masses of epididymal, retroperitoneal, and inguinal adipose depots), and liver (Fig. 1H). Importantly, HFHSu mice featured higher body weight gain, but not adiposity, than HFD fed mice (Fig. 1B and H). Furthermore, HFHSu mice had a smaller cumulative high‐fat intake and, consequently, free fatty acids intake during the 8 weeks of feeding (Fig. 1D), but higher caloric intake than HFD‐fed mice, because of 20% sucrose drinking (Fig. 1C).

Along with obesity, intake of both HFD and HFHSu impaired glucose homeostasis as evidenced by increased fasting glycemia (Fig. 1F) and insulinemia (Table 1), and severe glucose intolerance as illustrated by the higher glucose excursion (Fig. 1E), and area under the curve in the glucose tolerance test (Fig. 1G).

**Table 1:** Plasmatic parameters

	Control	HFD	HFHSu
Fasting insulin (mg dL^−1^)	47 ± 7	472 ± 99*	427 ± 75*
Postprandial insulin (pmol L^−^1)	2179 ± 216	2207 ± 420	1093 ± 361*, +
Leptin (ng dL^−1^)	8086 ± 1129	17 343 ± 2062*	16 102 ± 1401*
Resistin (ng mL^−1^)	4232 ± 626	4177 ± 841	4180 ± 378
Triacylglycerides (mmol L^−1^)	1.0 ± 0.2	2.2 ± 0.2*	2.2 ± 0.2*
Free fatty acids (mmol L^−1^)	0.2 ± 0.1	0.7 ± 0.1*	0.7 ± 0.1*
Interleukin‐6 (ng mL^−1^)	22 ± 4	21 ± 4	15 ± 3

Values are means ± SEM. Two‐way ANOVA. Significances (*P* < 0.05): *versus C; ^+^versus HFD.

Postprandial plasma levels of leptin, triglycerides, and free fatty acids were significantly elevated in HFD and HFHSu fed‐mice compared to C diet (Table 1). There were no significant changes in plasma levels of resistin and IL‐6 among different feeding regimens (Table 1). Thus, HFD was able to induce biochemical and metabolic alterations typical of those seen in obesity.

### High‐fat diet leads to different hemodynamic phenotypes and to autonomic imbalance

**Figure 2:**
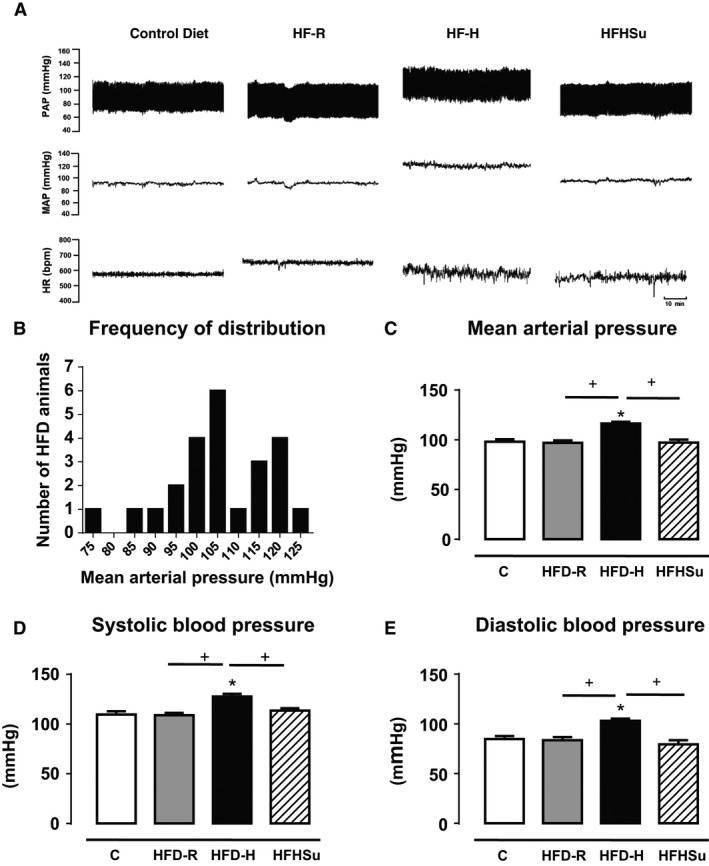
Cardiovascular parameters. Cardiovascular phenotype of mice fed with control (C, *n* = 8), high‐fat (HFD, *n* = 24) or high‐fat high‐sucrose (HFHSu, *n* = 11) classified in hypertensive high‐fat (HFD‐H, *n* = 9) or hypertension‐resistant high‐fat (HFD‐R, *n* = 15). (A) Representative traces of the pulsatile pressure (PAP, mmHg), mean arterial pressure (MAP, mmHg), and heart rate (HR, bpm) of one animal of each group; (B) Histogram of mean arterial pressure of HFD mice; (C) Basal mean arterial pressure (MAP mmHg), (D) systolic blood pressure (SBP, mmHg), (E) diastolic blood pressure (DBP, mmHg). (F) Heart rate (HR, bpm). *P < 0.05 versus C, ^+^ *P* < 0.05 versus group indicated. One‐way ANOVA with Newman–Keuls post hoc test.

Figure 2A depicts representative traces of pulsatile arterial pressure (PAP), MAP and HR from one conscious animal representative of each group fed with different diet. The bimodal distribution, depicted by two peaks in MAP in the histogram (Fig. 2B) shows that HFD mice were divided in two groups according to their hemodynamic phenotype: (1) obese hypertensive mice (HFD‐H) with distribution peak in 120 mmHg (Fig. 2B) and average value of MAP baseline of 114 ± 4 mmHg (Fig. 2C), and (2) obese hypertension‐resistant mice (HFD‐R) with distribution peak in 105 mmHg (Fig. 2B), and average value of MAP baseline of 97 ± 2 mmHg (Fig. 2C). Importantly, increase in MAP was dependent of elevations in both systolic and diastolic blood pressures (Fig. 2D and 2E). There were no differences in HR among groups (Fig. 2F). In contrast to HFD, obesity induced by the intake of a HFHSu was not associated with changes in MAP (HFHSu: 96 ± 4 mmHg) when compared with C group (98 ± 3 mmHg, Fig. 2C). No significant changes were observed in the HR among the groups (data not shown).

**Figure 3:**
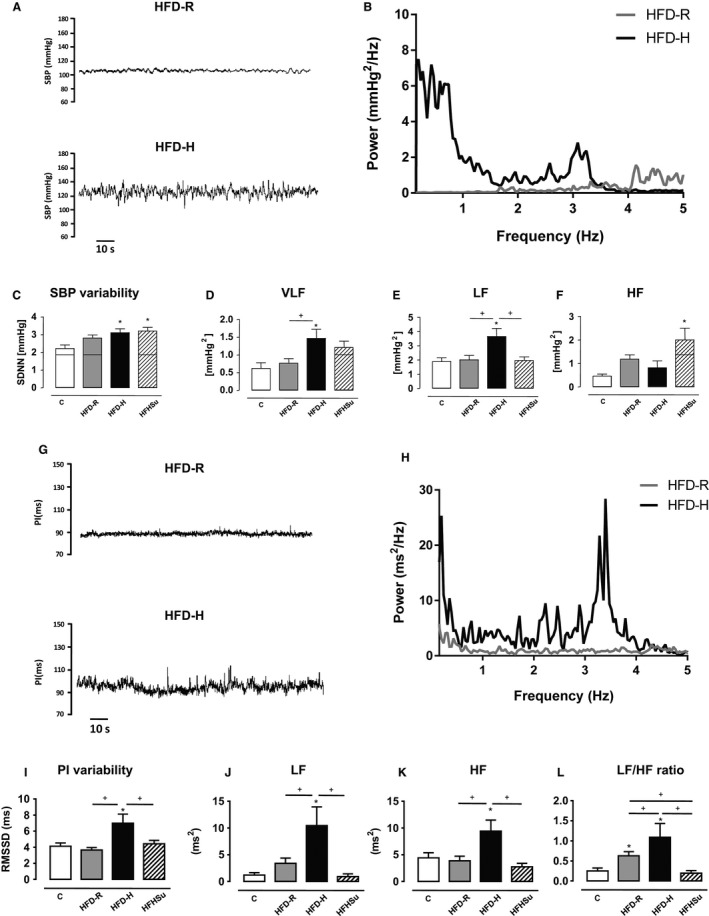
Power spectral analysis of the systolic blood pressure (SBP) and pulse interval (PI) variability. (A) Representative traces of SBP variability in HFD‐R and HFD‐H of one animal of each group; (B) spectra of SBP in HFD‐R and HFD‐H mice; (C) Analysis of the total variability in SBP (SDNN); (D) very low‐frequency component (VLF); (E) low‐frequency component (LF); and (F) high‐frequency (HF) of SBP. (G) Representative traces of PI of HFD‐R and HFD‐H mice in one animal of each group; (H) spectra of PI in HFD‐R and HFD‐H mice; (I) Total PI variability analysis represented by root mean square of successive differences (RMSSD), (J) low‐frequency component, (K) high‐frequency component, and (L) autonomic balance (LF/HF ratio) to the heart. *P < 0.05 versus C, ^+^ *P* < 0.05 versus group correspondent. One‐way ANOVA with Newman–Keuls post hoc test. C (*n* = 8), HFD‐R (*n* = 15) and HFD‐H (*n* = 9), HFHSu (*n* = 11).

Spectral analysis of cardiovascular variabilities was performed in the same group of animals. Regarding SBP variability (Fig. 3A), power spectral analysis (Fig. 3B) and the SDNN values displayed by HFD‐H mice were higher than those of chow‐fed mice (Fig. 3C) as evidenced by the increased VLF (Fig. 3D) and LF (Fig. 3E) components, which represent hormonal and sympathetic modulation of vessels, respectively. HFHSu also featured higher SBP variability (Fig. 3C) than C group, which, in contrast to HFD‐fed mice, was due to the increase in the HF component (Fig. 3F).

As evaluated by the traces and power spectra of PI variability (Fig. 3G and H, respectively), HFD‐H mice have shown a higher RMSSD values (Fig. 3I) in HFD‐H mice when compared with HFD‐R and C diet mice caused by increased LF (Fig. 3J) and HF spectral power (Fig. 3K). As a consequence, the LF/HF ratio, an index of sympatho‐vagal balance, was higher in HFD‐H group when compared with HFD‐R and C group (Fig. 3L).

As we have performed a direct blood pressure measurements in conscious freely moving mice, it is important to state that the effects of 1 day post surgical procedures were compared with 4 days of recovery to exclude stress surgery responses in arterial pressure and in autonomic balance (Table 2). There were no differences in the cardiovascular and autonomic parameters between 1 and 4 days recovery. This results suggest that this bimodal distribution in MAP seen in the group HF was not a stress response related.

**Table 2:** Blood pressure (MAP mmHg) and autonomic balance levels compared among animal′s group that underwent 1 and 4 days of recovering after blood vessels surgical procedures

	Groups			
	C	HFD‐R	HFD‐H	HFHSu
MAP (mmHg)				
1 day	98.2 ± 2.5	97.1 ± 2.3+	114.3 ± 3.4*	96.26 ± 3.7+
4 days	100.1 ± 3.6	105.2 ± 1.1+	122.8 ± 1.2*	90.9 ± 4.6+
LFsbp				
1 day	1.9 ± 0.3	2.0 ± 0.3+	3.7 ± 0.6*	2.0 ± 0.3+
4 days	1.5 ± 0.9	2.1 ± 0.3+	4.0 ± 1.1*	1.5 ± 0.2+
LF/HF				
1 day	0.25 ± 0.07	0.63 ± 0.10+	1.10 ± 0.35*	0.35 ± 0.19+
4 days	0.19 ± 0.05	0.66 ± 0.05+	2.18 ± 0.12*	0.5 ± 0.17+

Values are means ± SEM. Two‐way ANOVA. Significances (*P* < 0.05): *versus C; ^+^versus HFD‐H.

### CART mRNA level is higher in the hypothalamus of hypertensive obese mice

**Figure 4:**
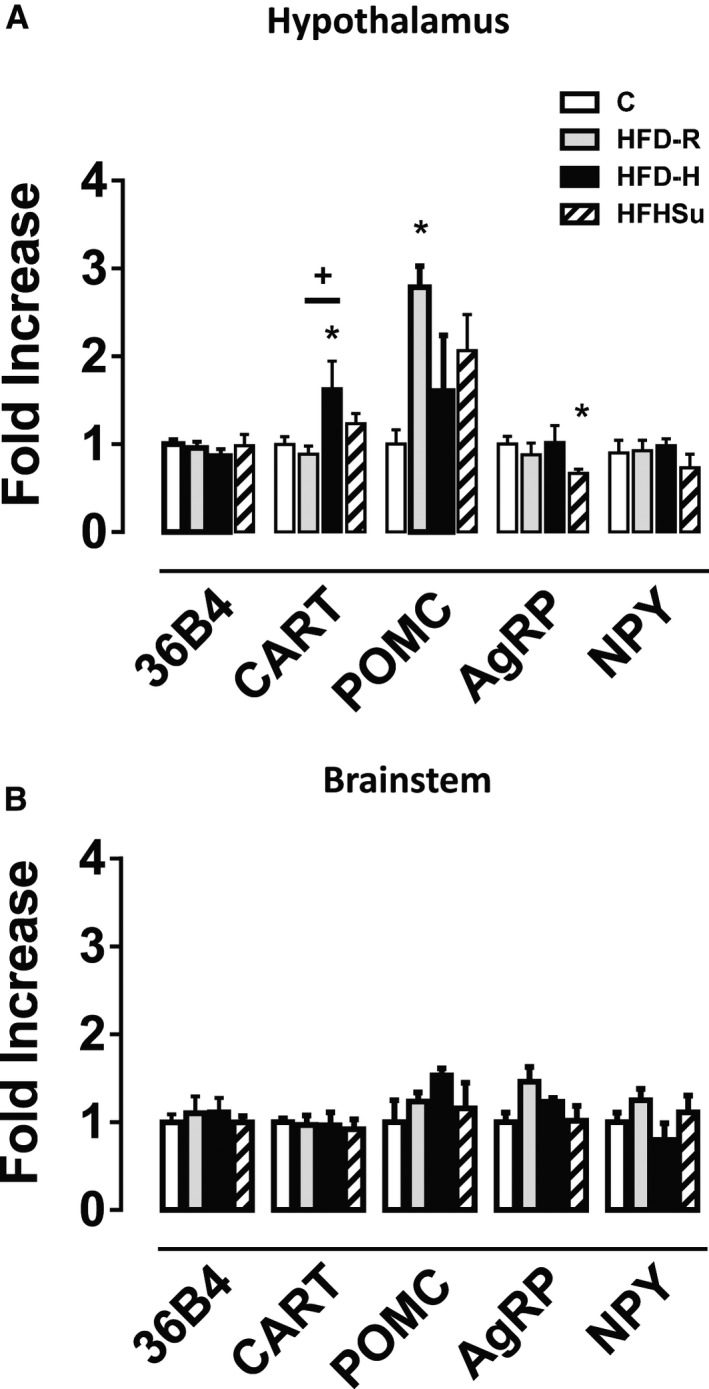
Gene expression of hypothalamic and brainstem neuropeptides. (A) Hypothalamic gene expression of the reference gene 36B4, CART, POMC, AgRP, and NPY. (B) Brainstem gene expression of 36B4, CART, POMC, AgRP, and NPY of C (*n* = 6), HFD‐R (*n* = 6), HFD‐H (*n* = 7), and HFHSu mice (*n* = 10). *P < 0.05 versus C, ^+^ *P* < 0.05 versus HFD‐R; One‐way ANOVA with Newman–Keuls post hoc test.

We next investigated whether the above‐described cardiovascular changes were associated with alterations in mRNA expression of neuropeptides involved in the regulation of food intake and energy expenditure within hypothalamus and brainstem in the same group of animals. As depicted in Figure 4, CART mRNA level was significantly increased in the hypothalamus, but not brainstem of HFD‐H mice (*n* = 7) when compared with HFD‐R (*n* = 6) and C group (*n* = 6, Fig. 4A). In contrast, POMC gene expression was higher in the hypothalamus, but not brainstem of HFD‐R when compared with HFD‐H and C diet mice (Fig. 4A). mRNA levels of AgRP and NPY in the hypothalamus and brainstem were not affected by any diet and/or hypertension (Fig. 4A and B).

### HFD‐fed‐mice have higher number of CART‐positive neurones in the dorsomedial hypothalamus

**Figure 5:**
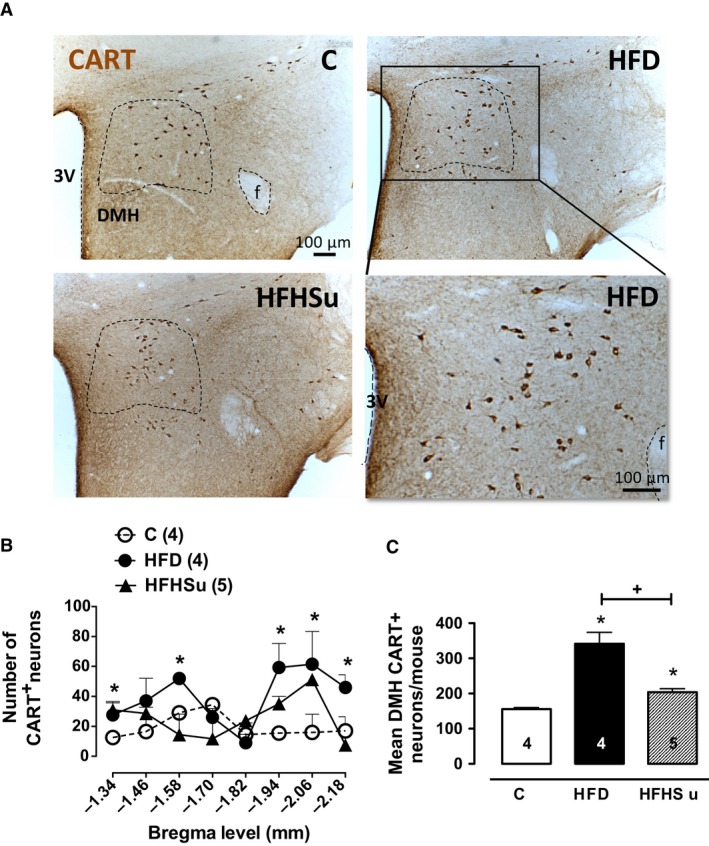
CART‐positive (CART^+^) neurones are higher in DMH of HFD mice. (A) Photomicrographs in light field at the DMH level of C (*n* = 4), HFD (*n* = 4), and HFHSu (*n* = 5) mice depicting the CART immunoreactive neuronal cell bodies. (B) Rostrocaudal distribution of CART^+^ in DMH from the bregma level among the groups. (C) Total number of CART^+^ neurones throughout the DMH. Fornix (f), third ventricle (3V). *P < 0.05 versus C, ^+^ *P* < 0.05 versus HFD; One‐way ANOVA with Newman–Keuls post hoc test.

**Figure 6:**
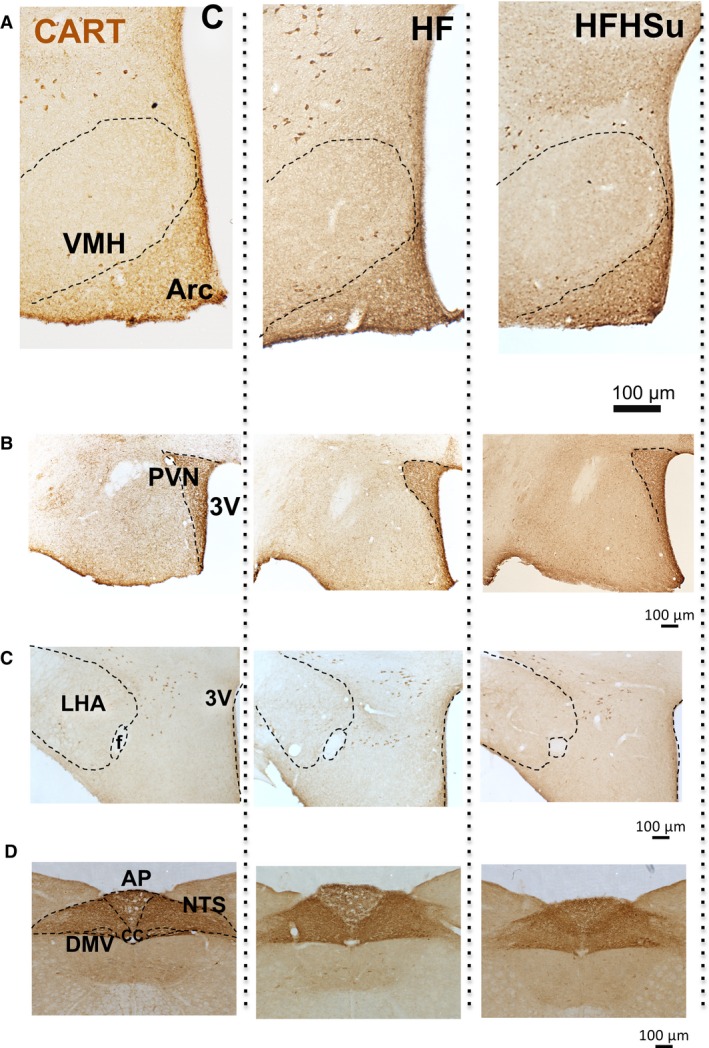
Immunoperoxidase reaction for CART at others hypothalamic and brainstem nuclei. Photomicrographs in light field 20× magnification at the DMH level of C (*n* = 4), HFD (*n* = 4), and HFHSu (*n* = 5) mice depicting the CART immunoperoxidase reaction at (A) ventromedial nucleus of hypothalamus (VMH), (B) paraventricular nucleus of the hypothalamus (PVN), (C) lateral hypothalamic area (LHA), (D) nucleus of the solitary tract (NTS). Fornix (f), third ventricle (3V), arcuate nucleus of hypothalamus (Arc), dorsal motor vagus nucleus (DMV).

Because CART gene expression was higher in the hypothalamus of HFD‐H mice, we next performed an immunoperoxidase reaction to determine in which hypothalamic nuclei would exhibit higher levels of CART. As depicted in Figure 5A, we found higher number of CART‐positive neurones counted in hypothalamic slices every 120‐*μ*m interval in the DMH of HFD‐fed mice when compared with compared to HFHSu and C groups (Fig. 5C). This higher number of CART‐positive neurones at the DMH were rostro‐caudally located approximately at the position 1.58 mm posterior to bregma and between 1.94 mm posterior to bregma until 2.18 mm posterior to bregma (Fig. 5B). We have not found any CART‐positive neuronal cell body, only fibers immunostaining, at other hypothalamic nuclei level, such as ventromedial nucleus of hypothalamus (VMH, Fig. 6A), paraventricular nucleus of hypothalamus (PVN, Fig. 6B), lateral hypothalamic area (LHA, Fig. 6C), and in the brainstem at the NTS (nucleus of tract solitary) level (Fig. 6D).

## Discussion

We investigated herein whether diet‐induced obesity is associated with autonomic imbalance, hypertension, and changes in hypothalamic and brainstem neuropeptide levels. Through the evaluation of cardiovascular parameters and hypothalamic gene expression in conscious, freely moving mice our main findings are as follows: (1) HFD and HFHSu induced obesity in mice, but only HF diet regimen was associated with hypertension; (2) HFD displayed two hemodynamic phenotypes in mice namely hypertension and resistant to hypertension; (3) Hypertensive HFD‐fed mice exhibit autonomic imbalance and increased CART mRNA levels in hypothalamus, but not in brainstem and CART‐positive neurones at the DMH. A relevant point in this study is that we were able to study an integrative association of gene expression of neuropeptides with hemodynamic and autonomic control of conscious freely moving mice model of obesity. This approach eliminated the influence of anesthesia on the neurotransmission of autonomic brain nuclei that regulated the blood pressure levels (Holscher et al. 2008).

Feeding mice with a high‐fat diet is known to produce biochemical and metabolic changes, which are commonly found in human obesity (Buettner et al. 2007). Corroborating these studies, here we have demonstrated that HFD or HFHSu intake for 8 weeks induced obesity, as evidenced by the increased body weight and adiposity, and impaired glucose homeostasis, as indicated by the increased fasting glycemia, insulinemia and severe glucose intolerance. Such similar metabolic alterations are observed in human obesity, which suggests that these animal model are suitable for investigating mechanistic links between obesity and hypertension. As for the body weight we have observed that HFHSu group had an increased gain compared to HFD group, besides consumed less amount of high‐fat diet. We believe that the reason of this results is the total caloric intake, considering also the sucrose intake, that is increased in the HFHSu group comparing with the HF group. Since only the HFD group became hypertensive, our data imply that diet type per se is important, rather than obesity, in hypertension development.

One interesting caveat is that blood glucose was high in both HFD and HFHSu although postpradial insulin was lower in HFHSu than in HFD. We do not know what is the precise mechanisms underlying the reduced postprandial insulin levels featured by HFHSu. Plasma insulin levels represent the balance between insulin secretion by pancreatic beta cells and insulin clearance. HFHSu mice also featured lower insulin levels 30 min after an intraperitoneal glucose injection in the glucose tolerance test (data not shown), indicating that HFHSu mice probably have a defect in insulin secretion rather than in clearance. Further experiments analysis of glucose‐induced insulin secretion by isolated islets in vitro are required to confirm this hypothesis.

Interestingly, in mice fed with a HFD for 8 weeks two different hemodynamic phenotypes were noticed: (1) some animals become hypertensive; and (2) others were resistant to high‐fat‐induced hypertension. Therefore, this is an interesting animal experimental model to study obesity‐related hypertension because allows to compare whether neuropeptides gene expression would be distinct between these two hemodynamic phenotypes. Beside of this, HFHSu did not induced hypertension, and particularly this group of mice had a smaller cumulative high‐fat intake and, consequently, free fatty acids intake during the 8 weeks of diet, because of the addition of sucrose solution in the regimen. This can be a mechanism to explain that this group did not developed hypertension, which is the aim of forthcoming studies. The reason why mice with identical genetic background and fed with the same regimen display different cardiovascular outcomes in response to diet‐induced obesity remains unknown.

The mechanisms underlying the development of obesity‐induced hypertension are multifactorial and complex. Several studies indicate that, among neurohumoral, renal, and vascular factors, overactivation of the sympathetic nervous system contributes to the etiology of hypertension in obese humans and animal models (Esler 1995; Reaven 2002; Hall 2003; Grassi et al. 2004; Bergman et al. 2007; Hall et al. 2010). Thus, one aim of our study was to evaluate the role of the autonomic balance on the hypertension secondary to high‐fat diet‐induced obesity. In this sense, the spectral analysis of arterial pressure and heart rate variability, an important noninvasive tool previously validated to evaluate the physiological influence of autonomic balance in control of cardiovascular parameters. Our main findings indicate that HFD‐induced hypertension is associated with an increase in the LF component of SBP and PI, which is an index of vascular sympathetic outflow and cardiac sympathovagal balance, respectively (Thireau et al. 2008). Our findings differ from previous studies, which have shown that hypertension was associated with an increase in LF of PI, but not LF of SBP variability (Williams et al. 2003) in HFD‐fed mice for 15 weeks. This discrepancy between studies might be due to different control diet used or mice age at the beginning of the protocols, which were younger (5 weeks old) than we used in our study (10 weeks old).

Further supporting the involvement of sympathetic hyperactivity in obesity‐associated hypertension are the previous findings that: (1) pharmacological blockade of adrenergic activity lowers blood pressure to a greater extent in obese than in lean subjects (Wofford et al. 2001; D'Angelo et al. 2006); (2) renal denervation markedly decreases sodium retention and hypertension in obese animals (Kassab et al. 1995); (3) administration or chronic treatment with *α*‐ and *β*‐adrenergic receptor antagonists or clonidine, a drug that stimulates central *α*_2_ adrenergic receptors, reduces SNS activity and prevents the increase in blood pressure in dogs fed with HF diet and in hypertensive obese patients (Wofford et al. 2001; Wofford and Hall 2004; Hall et al. 2010).

We hypothesized that HFD would lead to changes in gene expression of neuropeptides in the central nervous system that could be correlated with obesity‐induced hypertension and autonomic imbalance. Indeed, we observed that HFD elicits a significant increase in the CART mRNA expression in the hypothalamus of HFD hypertensive rats when compared with HFD resistant and control diet group.

Cocaine‐ and amphetamine‐regulated transcript is a neuropeptide involved in different behaviors and processes, such as reward, addiction, feeding, regulation of body weight, energy expenditure, and hypothalamic‐pituitary‐adrenal axis activation in stress (Rogge et al. 2008). Immunohistochemical studies reveal a neuronal network of CART positive‐neurones along the sympathetic‐adrenal (Fenwick et al. 2006) and the sympathetic‐cardiac axis, including the rostral ventrolateral medulla (Burman et al. 2004), preganglionic sympathetic neurones in intermediolateral column of the spinal cord (Elias et al. 1998; Fenwick et al. 2006), chromaffin cells in the adrenal medulla (Koylu et al. 1997) and in the intracardiac ganglia in rats (Richardson et al. 2006).

Intracerebroventricular, intrathecal, or intracisternal CART administration in anesthetized rats and conscious rabbits evokes a significant increase in blood pressure and HR (Matsumura et al. 2001; Hwang et al. 2004; Scruggs et al. 2005). CART has been shown to excite directly neurones within the central neural pathways that control the SNS directly or indirectly by potentiating the action of glutamate, via NMDA receptors (Hsun Lin et al. 2005; Chiu et al. 2009, 2010).

Among several neuromodulators involved in activation of the SNS, one of the most studied in obesity is leptin, an adipokine secreted by adipocytes proportionally to the degree of adiposity. High levels of leptin could explain, at least in part, the obesity‐induced hypertension and renal sympathetic hyperactivity of humans (Rahmouni et al. 2002; Eikelis et al. 2003) and mice (Simonds et al. 2014). Thus, leptin would be a link between the excess of adiposity and sympathoexcitation (Rahmouni et al. 2005). Leptin regulates energy homeostasis by acting on hypothalamic neuronal circuits to reduce calorie intake and increase energy expenditure (Friedman 2009). In the Arc nucleus leptin inhibits NPY/AgRP (Schwartz et al. 1996), but activates POMC/CART neurones (Elias et al. 1998; Cowley et al. 2001). Additionally, leptin stimulates CART/NPY neurones within the DMH, that project to the PVN (Lee et al. 2013a), in diet‐induced obesity condition (Lee et al. 2013b).

Humans with loss‐of‐function mutations in leptin or leptin receptor have low blood pressure, despite being obese (Simonds et al. 2014). Importantly, it was recently shown that leptin increases blood pressure in conditions of diet‐induced obesity by acting on DMH neurones (Simonds et al. 2014). Indeed, blockade of leptin action with a specific antibody, pharmacological antagonists, or inhibiting the activity of leptin receptor‐expressing neurones in the DMH lead to a fall in the MAP in diet‐induced obese mice, such effect that was reversed through re‐expression of the leptin receptors in the DMH (Simonds et al. 2014).This study demonstrates that leptin is coupled to obesity‐induced blood pressure changes in humans and mice (Simonds et al. 2014).

The mechanisms, however, through which leptin acts at the DMH promoting obesity‐associated hypertension are still unknown. We could argue that perhaps leptin have distinct actions in HFD hypertensive animals compared to hypertension‐resistant animals. Noteworthy, HFHSu mice and HFD hypertension‐resistant mice, despite having elevated levels of leptin, did not develop hypertension, or featured elevated hypothalamic CART mRNA expression and CART‐positive neurones in DMH when compared to C diet. These data imply that the leptin circulating levels *per se* do not seem to be related to CART‐associated hemodynamic changes at the DMH level. In obese humans (Haynes et al. 1999; Rahmouni et al. 2002) and mice (Simonds et al. 2014) high levels of leptin elicits a renal sympathetic hyperactivity and hypertension, such effect not seen in leptin‐deficient animals (Simonds et al. 2014). Take together, these findings suggest that is not the leptin circulating levels that determine hypertension and autonomic imbalance, but instead, is the way how this hormone acts in distinct neurones in the hypothalamus. It has been showing elsewhere that obese high‐fat fed animals have an increase in plasmatic leptin levels, and this hormone has a reduced sensitivity to activate its receptors, not only in the periphery but also centrally, leading to a condition of leptin resistance (Lin et al. 2000). In this study, we can suggest that a hypothalamic leptin resistance could interfere in the leptin‐stimulated production of CART. In this sense, CART may be an important neuropeptide involved in the leptin actions on the cardiovascular system, because peripheral administration of leptin was shown to increase CART expression within the hypothalamus, including the DMH (Elias et al. 1998; Kristensen et al. 1998; Lee et al. 2013b).

Despite the structure and effects of CART have been known for at least 15 years, CART receptor has not been identified, sequenced and cloned yet, and for this reason there is no pharmacological antagonist available yet limiting progress in this area of research. There is one study about CART receptors, showing that CART had an low nonspecific binding in cell cultures (Maletínská et al. 2007). The lack of selective antagonists to block CART receptor could contribute positively to our studies to confirm the CART functional role in the hypertensive‐obese animals. Therefore, it is plausible to assume that CART could be directly related to the hyperactivity of the sympathetic nervous system in obesity‐associated hypertension, but this assertion still waits further investigation.

Collectively, we can conclude that HFD‐induced hypertension and autonomic imbalance are associated to an upregulation of CART levels in the DMH of mice. On the other hand, mice on an HFHSu diet do not have an elevated blood pressure, autonomic imbalance, or increased CART gene expression and peptide content in DMH. Further studies are, however, required to evaluate the possible causality between these phenotypes.

## Perspectives

Chronic high‐fat intake, from the translational point of view, may bring important insights into the mechanisms underlying hypertension secondary to obesity. This study highlights the relevance of CART in the sympathovagal imbalance to the heart and cardiovascular alterations associated with obesity. Thus, a better understanding of the underlying mechanism of CART increase in DMH in hypertension would help to uncover new pharmacological and potentially nonpharmacological therapies to overcome the common maladies caused by hypertension secondary to obesity.

## Conflicts of Interest

None declared.
